# A review of the pangenome: how it affects our understanding of genomic variation, selection and breeding in domestic animals?

**DOI:** 10.1186/s40104-023-00860-1

**Published:** 2023-05-05

**Authors:** Ying Gong, Yefang Li, Xuexue Liu, Yuehui Ma, Lin Jiang

**Affiliations:** 1 Laboratory of Animal Genetics, Breeding and Reproduction, Ministry of Agriculture, Institute of Animal Sciences, Chinese Academy of Agricultural Sciences (CAAS), Beijing, 100193 China; 2 National Germplasm Center of Domestic Animal Resources, Ministry of Technology, Institute of Animal Sciences, Chinese Academy of Agricultural Sciences (CAAS), Beijing, 100193 China; 3 Centre d’Anthropobiologie et de Génomique de Toulouse, Université Paul Sabatier, 37 allées Jules Guesde, Toulouse, 31000 France

**Keywords:** Breeding, Domestic animals, Pangenome, Structural variations

## Abstract

As large-scale genomic studies have progressed, it has been revealed that a single reference genome pattern cannot represent genetic diversity at the species level. While domestic animals tend to have complex routes of origin and migration, suggesting a possible omission of some population-specific sequences in the current reference genome. Conversely, the pangenome is a collection of all DNA sequences of a species that contains sequences shared by all individuals (core genome) and is also able to display sequence information unique to each individual (variable genome). The progress of pangenome research in humans, plants and domestic animals has proved that the missing genetic components and the identification of large structural variants (SVs) can be explored through pangenomic studies. Many individual specific sequences have been shown to be related to biological adaptability, phenotype and important economic traits. The maturity of technologies and methods such as third-generation sequencing, Telomere-to-telomere genomes, graphic genomes, and reference-free assembly will further promote the development of pangenome. In the future, pangenome combined with long-read data and multi-omics will help to resolve large SVs and their relationship with the main economic traits of interest in domesticated animals, providing better insights into animal domestication, evolution and breeding. In this review, we mainly discuss how pangenome analysis reveals genetic variations in domestic animals (sheep, cattle, pigs, chickens) and their impacts on phenotypes and how this can contribute to the understanding of species diversity. Additionally, we also go through potential issues and the future perspectives of pangenome research in livestock and poultry.

## Introduction

Since the beginning of the genomic age, individual reference genomes have become the basis for understanding genetic variation in organisms. By aligning sequencing reads with a reference genome to identify the fragment variants such as single nucleotide polymorphisms (SNPs) and small insertions and deletions (indels), new insights into biological genetic diversity, population history and genome-based breeding can be obtained [1–6]. However, in addition to SNPs and indels, genomic variation includes many long-fragment structural variants (SVs), such as copy number variations (CNVs), rearrangements and presence/absence variants (PAVs) [7–9]. Although representing only 0.2% of genomic variation types, SV can still affect 4%–12% of coding genes by changing gene dosage and interfering with gene function in humans [10–12]. Past studies have found that there are limitations in the alignment of one single reference genome as well as in sequencing techniques, which reduces the detection efficiency of those sequences that are significantly different from the reference genome and from large segments of variants longer than 50 bp [13, 14]. In addition to this problem, the complexity of SV computational models, the mixing of repeats, and the errors generated by sequencing make SV the most difficult type of variant to capture by short-read sequencing [12, 15, 16].

The maturation of the high-throughput sequencing technology has caused a surge in genome sequencing in a number of different species. Massive amounts of the available genomic data, together with the flaws in past approaches to characterizing the genetic diversity of species, pangenomes (collections of all nonredundant DNA sequences in a species or population) have gradually become new reference coordinates for genomics research. The first pangenomes were implemented in bacteria, and the variable genomic components were related to bacterial virulence genes and to some nonessential biological pathways, which were important for subtyping different bacteria and for developing vaccines [17]. To date, pangenomes have been studied extensively in both bacteria and viruses. However, the progress has been slower for eukaryotes, due to the complexity of their genomes and the difficulties encountered when compiling complete pangenomes [13, 18]. Moreover, due to the continuous development of third-generation sequencing technology (TGS) in recent years, the integrity of the assembly and annotation of the complex genomic regions and the precise SV detection capability have been improved [19–21]. This will provide a strong boost to the field of eukaryotic pangenome research.

In this review, we briefly outline the origin of the pangenome concept and summarize our current knowledge of the pangenome as a new reference standard for mining the biological genetic variation resources. Then, we give some examples to illustrate the key findings from these studies related to the development of the eukaryotic pangenome and to the expression of pangenetic effects on biological evolution and adaptability. Next, we discuss how to use pangenomes, together with advances related to the domestic animal pangenome, to identify and analyse new functional sequences and genetic variations, and to improve breeding by analysing the effects of genetic variations on traits. Finally, we discuss the challenges and future perspectives of using pangenomes to advance gene and sequence function analysis in livestock and poultry.

## Extensive sequencing data and high-quality components are the foundation of pangenome construction

**Fig. 1:**
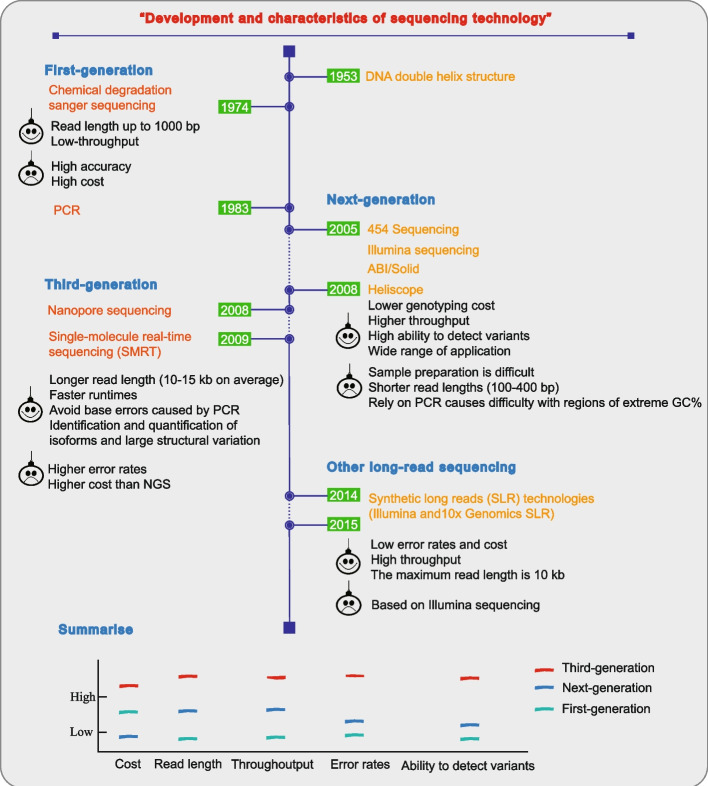
The development and features of sequencing technology. The timeline in the graphic depicts the development of genome sequencing technologies through time. While this was going on, a comparison and summary of the traits, benefits, and drawbacks of sequencing from the first to the third generation was made. The examination of complicated genome structure and variation is made easier by TGS’s rapid, real-time, and ultra-long read length

Advances in sequencing technology provide a database and technical support for the research on eukaryotic pangenomes (Fig. 1). Although the next-generation sequencing (NGS) technology created by Illumina has greatly improved the single throughput and the ability to detect genomic variation in a high throughput way [20, 22]; however, NGS has the major defect of short read length, and using PCR during the process can lead to the base bias, which reduces the ability to detect complex genomes. By contrast, TGS, as represented by PacBio technology, has a read length of up to 80 kb on a high-throughput basis, greatly improving the detection and analysis capabilities of complex regions and large SVs of the genome [19, 20, 23]. Although TGS has the advantages of long read length and high accuracy, its application is currently constrained by its expensive cost and dearth of bioinformatics data analysis software [20]. In addition, a long-read sequencing technology, synthetic long read (SLR), whose sequencing cost and error incidence are lower than those of TGS, is widely used for cell sequencing [20] . These technologies have enabled many individual genomes of species to be sequenced. To date, this has yielded over 1.2 million genomes and a vast amount of the genomic data.

**Fig. 2:**
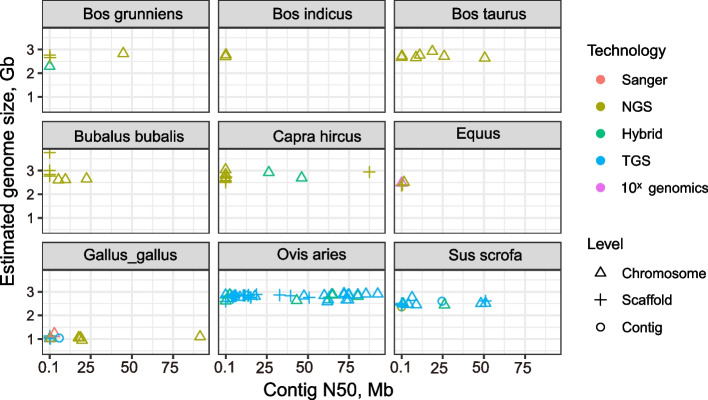
Current research status of major livestock and poultry genomes. Colors represent sequencing platforms where red (Sanger), olive green (NGS), green (Hybrid), blue (TGS) and purple (10× genomics); the assembly level are indicated in angular (chromosome), plus (scaffolds) and circle shapes (contig)

With the advent of these technologies, a variety of reference genomes have been successively unfolded. Taking domesticated animals as an example, the chromosome-scale assemblies such as ARS1 [24], Saanen_v1 [25], Sscrofa11.1 [26], ARS-UCD1.2 [27] have been produced. And the contig N50 of multiple components reached more than 20 Mb and a maximum above 92 Mb, with extremely high genomic continuity and integrity (Fig. 2). The reference genomes can be used for coordination, through comparative analysis of large sequencing data, to deeply study the scientific issues of origin, domestication, disease resistance, and biological adaptability of different species. A series of genomic variations and molecular markers related to major traits of economic value have been identified. These results provide a reliable data support and a theoretical basis for understanding the mechanisms of occurrence, the prevention of related diseases and the improvement of varieties. Moreover, it also has given birth to the 1000 goat genomes project, the bovine genome sequencing project and the ruminant genome project, which have further promoted the research of functional genomics in livestock species [28–30].

To address the deluge of data from whole-genome wide sequencing and analysis, genomic databases such as the NCBI databases, the goat genome database, the bovine genome database and variation databases such as GGVD [31], PigVar [32] and BGVD [33] have been established. Overall, the development of sequencing technology has greatly promoted the volume of gene sequencing data available for each species, enabling genome research related to diversification and deep refinement and facilitating the development of pangenome research in eukaryotes.

## The pangenome concept originated in the comparative analysis of bacterial genomes

Since the advent of sequencing technology, many different bacterial genomes have been generated. In theory, one or more of these genomes can be used to describe a species, but the question of how many genomes were needed to fully describe a bacterial species has yet to be resolved. In 2005, Tettelin et al. [17] explored this issue by comparing the genomes of eight different bacterial strains and were the first to propose the concept of the pangenome to define a certain bacterial species. This pangenome contained a core genome (genes present in all the bacterial strains) and a nonessential genome (genes absent in one or more strains and genes unique to each strain). The core genome included most of the housekeeping genes, which remain unknown for other genomes assembled later. These results indicated that there are additional implications in analysing the Group B *Streptococcus* pangenome. Since this pioneering work, bacterial pangenomes have been studied from different perspectives, such as those related to the number of genomes/strains, higher taxa and mathematical prediction models [34–42].

The bacterial pangenome indicates that there is limited information available for comparative analysis of genetic variation between several individual genomes. Therefore, explaining the complex diversity and biological properties of a species requires more genomic information. Unlike the genes of the core genome, the nonessential genes that are present to enrich the diversity of bacterial species and participate in the biochemical pathways and functions that are not essential for bacterial growth, often resulting in selective advantages, such as adaptation to different ecological environments, antibiotic resistance or colonization of a new host [35, 43]. This suggests that a large number of variable new genes of value and research significance can be discovered by means of pangenome analysis.

## Four classical methods for constructing the pangenome

**Fig. 3:**
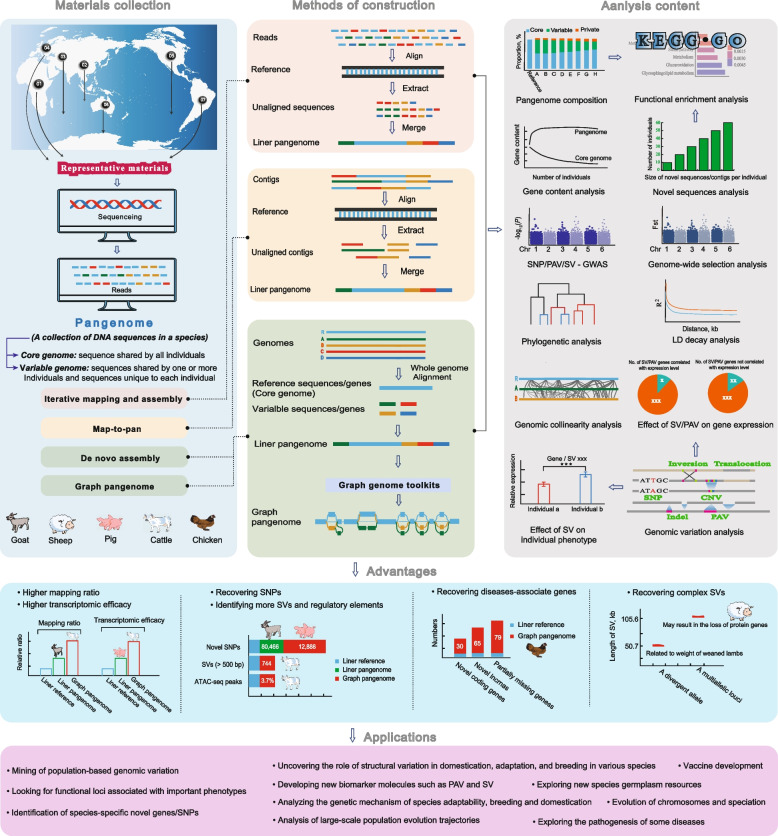
An overview of the construction methods, study areas, benefits, and applications of pangenome. The top panel explains the concept of a pangenome, sample collection, four pangenome construction approaches, and lists the analytical materials that go into creating one. The advantages of the pangenome over the linear reference genome are demonstrated in the middle section, primarily utilizing instances from domesticated animals. The pangenome’s primary uses are detailed in the final panel. Briefly stated, the pangenome project uses NGS and/or TGS to first gather the genomic data of representative samples worldwide, and then uses various construction techniques to compare these data to the reference genome or do comparative genomic analysis. Following the development of the pangenome concept, the main emphasis is placed on the core/dispensable genome, novel sequences, and SVs, combining with GWAS and transcriptomics to examine the relationship between genes and function, biology, and adaptation. Using discovered variants to build a graphical pangenome, which has significant advantages in defining genomic characteristics [51, 52], is a clear emerging trend today

Currently, there are three main methods for establishing a eukaryotic pangenome (Fig. 3). The first method is called iterative mapping and assembly. First, NGS reads are mapped to the reference genome, and then sequences that are not aligned are extracted. These sequences are further used as a supplement to the reference genome, and then are used to build the pangenome (such as has been used to build the pangenomes of humans [44], *Gallus gallus* [45] and *Sorghum bicolor* L. [46]). The second strategy is known as map-to-pan. After obtaining the de novo assembled contigs for each sample, they are aligned to the reference genome to determine those nonreference sequences, which together with the reference genome constitute the pangenome of the species (such as in the pangenome of *Solanum lycopersicum* [47] and cutton [48]). Currently, these two methods are mainly based on previously generated short segment data and then on benchmarking a certain reference genome, and their detection efficiency, accuracy and ability to capture some specific SVs beyond the reference are not superior. The third is the de novo assembly approach, in which the genomes of each individual are de novo assembled and annotated, and then a comparative analysis is performed to identify different fragments or genes, which are defined as nonessential genes (such as in the pangenomes of *Sesamum indicum* [49] and *Sus scrofa* [50]). While this method can accurately classify SVs, it is difficult to obtain the assemblies from scratch for comparison due to the high cost, as well as assembly and annotation errors.

In recent years, a graph pangenome was constructed using bidirected variation graphs and Graph Genome toolkits [53, 54]. On the basis of de novo assemblies, taking into consideration the genomic location of variable sequences from haploid/inbred organisms or the fully haplotype-phased contigs (as branches), progressively added them to the reference genome to build the graphical pangenome [55, 56]. Several recent studies have emphasized the great potential of using such genome maps in improving the accuracy of read mapping and variant calling, reducing mapping bias, and identifying ATAC-seq peaks and variants that cannot be identified by linear genomes (such as those of soybean [57], rice [58], sorghum [59] and cattle [51]). These graphical genomes can highlight the positional relationship between sequences and give an accurate portrayal of species genomic diversity, which is a crucial step for fully mining population genomic resources.

Overall, each of the four approaches offers benefits and drawbacks (Table 1). In comparison, the first two methods are more suited for the analysis of short-read data sets, which can satisfy the needs of large-scale genomic data analysis. The latter two approaches offer distinct advantages in the precise mapping of important trait control genes and SVs because they pay more attention to the amount and quality of de novo genome assembly. Graphical pangenome has gained popularity in recent years due to its ability to precisely collect and present the spatial information of genetic variation in the genome.

**Table 1:** Comparison of four construction methods of pangenome

Feature	Iterative mapping and assembly	Map-to-pan	De novo assembly	Graph pangenome
Reference	Reference-based	Reference-based	Reference-free	Reference-free
Assembly level	Short reads	Contigs	Genomes	Genomes
Alignment approach	Iterative alignment	Genome-wide alignment		
Sequencing depth requirements	Low depth	Low depth	High depth	High depth
Scope of application	Large-scale group	Large-scale group	Few individuals	Few individuals
Sequence storage	Liner	Liner	Liner	Graphic entity
Computing resource requirements	Low	Large genomes are in high demand	Higher	Higher
Analysis of SVs	Only SNPS and indels can be identified	Interindividual CNVs	Large SVs	Some highly divergent SVs
Cost	Low	Medium	Higher	Higher
Limitation	The sequence fragment is too short to analyze CNV	Not suitable for scale analysis of complex genomes	The genome needs to be annotated, which would lead to bias	Visualizing storage is difficult and ambiguity may arise during sequence alignment

## Research focus and applications of the pangenome

The pangenome can complement the missing genetic information based on analysis of a single reference genome, unearth the hidden genetic variations, and demonstrate the true genetic diversity at the species level [51, 58, 60]. In addition, many studies have shown that the read mapping ratio, transcriptome alignment efficiency, and the call rate of some rare and large variants can be significantly increased by using the pan-genome as a reference [50–52]. In general, there are three main research aspects in pangenomics. We created a visualization of each study component (Fig. 3). The most basic research focus of the pangenome is the characterization of core and variable genomes. Specifically, this includes assessing pangenome size, core genome size, and core versus variable genome structure, as well as carrying out a composition comparison.

The process of identifying and genotyping variations is yet another crucial aspect. Combining phylogenetic analysis, genome-wide association studies (GWAS), and RNA-seq data to identify special variants, locate important functional genes, and investigate the influence of SV on gene differential expression. Based on SV sets, pangenomics can further explore the genetic mechanism behind chromosomal evolution, population genomic organization, and species domestication, enhancing the study of disease, target trait breeding, and functional biology. In addition to concentrating on genome sequence variation, an intriguing research topic is to explore the effects of genomic SVs, transposon, and chromatin structure changes on the evolution of regulatory networks between noncoding regulatory elements and homologous genes in combination with three-dimensional genomics. The study of the interaction between SV, chromatin rearrangement, and transcriptional regulation of corresponding genes will provide new insights into the structure and function of species genome non-coding regulatory regions [61].

Additionally, a crucial component of pangenome study is the examination of newly discovered genes’ biological functions. Pangenome can identify non-reference sequences that often belong to non-core genomes and may have important implications for the fitness of organisms [62]. Therefore, analyzing their distribution among individuals and the function of the included genes can provide a better understanding of the species’ adaptation to extreme environments.

We go into further detail about specific examples of pangenome applications in the eukaryotic and domestic pangenomics sections.

## Development of the eukaryotic pangenome

Eukaryotic pangenomes are different from prokaryotic ones because their genomes show large differences. Most bacterial genomes are made up of short protein-coding sequences of approximately 1,000 bp, while the genomes of eukaryotes are at least 10,000 times larger than a bacterial genome due to the presence of introns and intergenic regions [63]. Smaller genomes consist mainly of coding sequences, and when the genome exceeds 500 Mb, genes and intergenic sequences expand almost equally, with approximately 50% of exons considered to be largely negligible [63]. Therefore, to construct the pangenome of eukaryotes, we should take all DNA sequences within the genome into consideration for the pangenome to truly play the role of a reference object.

**Fig. 4:**
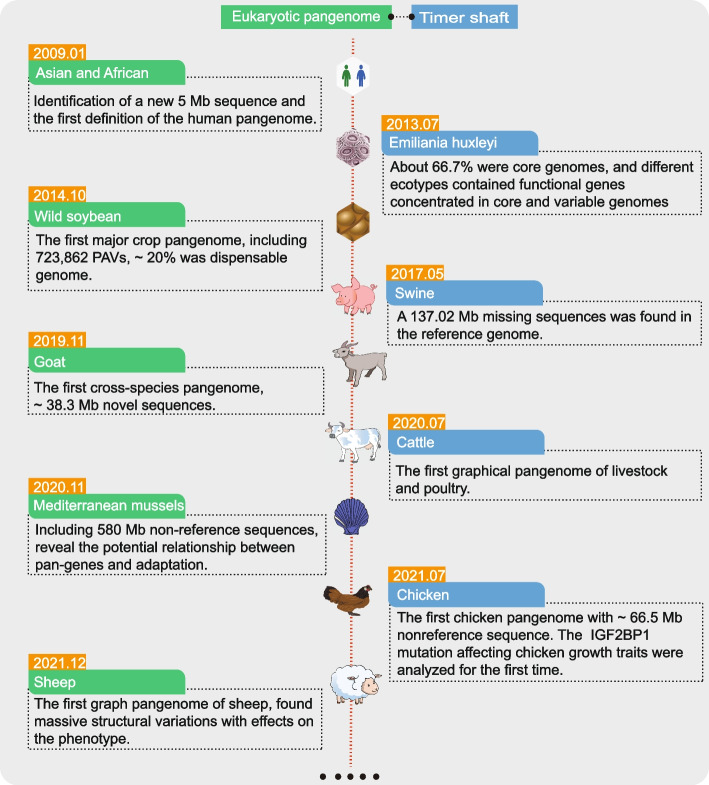
Eukaryotic pangenome development overview. This graph summarizes the key characteristics of these pangenomes, such as the core genome fraction and SV numbers, and briefly displays the publication dates of the first pangenomes of various species

Due to constraints such as sequencing technology, cost and genome complexity, eukaryotic pangenome research began later than prokaryotic pangenome. It was not until 2009 that pangenomic was being applied to human genomics studies based on the completion of the Human Genome Project [64] and multiple reference genome assemblies [65–68]. Pangenome studies of animals and plants have only gradually been carried out since 2013 (Fig. 4).

### Human pangenome analyses demonstrate a large number of non-reference sequences

**Fig. 5:**
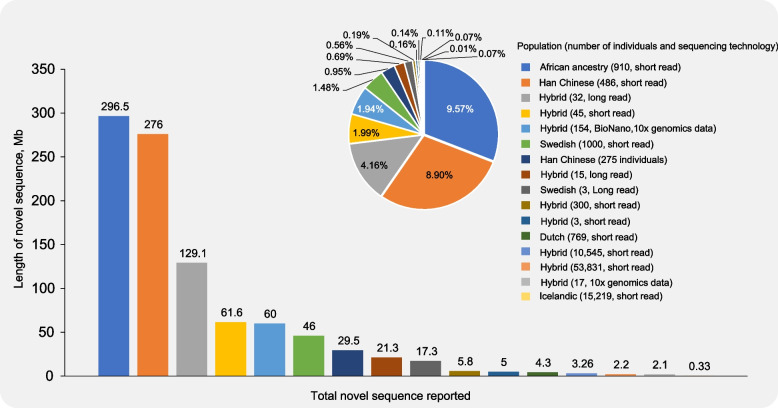
A review of recent research on the human pangenome. Bar and pie graphs show the number of novel sequences identified in published studies on the human pangenome and the proportion of such sequences in the human reference genome (GRCh38), respectively. The populations, subject counts, and sequencing techniques used in each study are displayed using colored annotations

The study of human pangenomics is a good example to verify that pangenome can effectively mine individual specific sequences and thus expand the range of existing reference genomes. In 2009, Li et al. [69] compared de novo assemblies in Asia and Africa and found approximately 5 Mb of specific sequences independent of the human reference genome. This study was the first to propose the concept of the ‘human pangenome’ (a nonredundant set of all DNA sequences in human populations). Subsequently, the human pangenome underwent more extensive research [70–74], and the number of novel sequences identified also increased (Fig. 5). For example, 296.5 Mb nonreference sequences were found in 910 African human genome comparisons [75], which was far more than the previously set of the new sequence size at the species level. In the pangenomic analysis of 486 Chinese people, 276 Mb of novel sequences were identified, and the average contained 46.646 Mb of common sequences (shared by at least 2 individuals). The common sequences were mainly distributed in the genomic regions with a high incidence of mutation and a low pathogenicity, which may be related to the changes in phenotypic adaptation of population to local environmental conditions [44].

### Plant pangenome studies suggest that many of the large structural variations affect biological traits and fitness

**Fig. 6:**
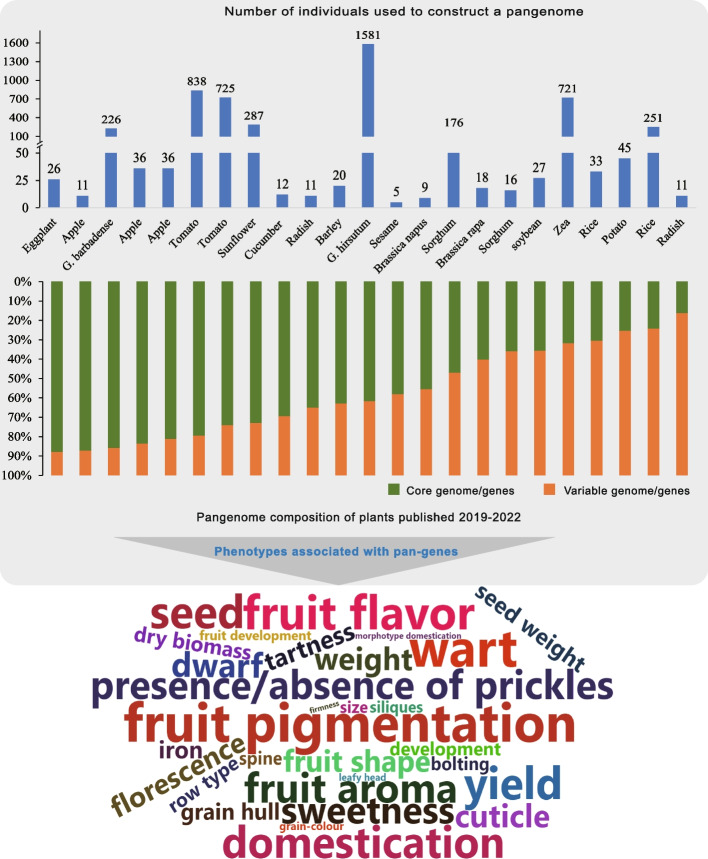
Plant pangenome summaries that have appeared in the previous four years. The multiple pangenome-building techniques are denoted by various colors. The top bar represents the number of individuals used to construct the pangenomes, and the bottom bar shows the ratio of core and variable genomes in the pangenomes

The development of plant pangenomes shows that using materials with different relatives, regions and phenotypes as research objects, pangenomics can comprehensively explore different types of SVs and promote the process of plant breeding (Fig. 6). The concept of the pangenome in plants was first mentioned in 2007 in the publication ‘Transposable elements and the plant pan-genomes’, which explained the role of transposers in the construction of the pangenome [76]. In 2014, Li et al. [77] published the first pangenome of plants by comparing seven soybean genomes. This pangenome is 30.2 Mb larger than the single genome, and a subset of specific genes and CNVs may cause changes in agronomic traits such as seed composition, flowering time, and organ size. With the development of long read sequencing and computer algorithms, the quality of gene sequencing and assembly has gradually improved, which further promotes the study of the plant pangenome. The relationship of the pangenome to disease resistance, flavour, and selective pressure, as well as to variants such as gCNVs, and PAVs, in crop agronomic traits has been explored in several species [60, 78–84]. This is a significant advance in plant pangenomic research that links plant phenotypes with large SVs (PAV-GWAS, CNV-GWAS and SV-GWAS), breaks the previous limitations of SNP-GWAS, and accelerates the understanding of the genetic basis of important traits in crops. For example, the 1179 Mb pangenome of the cultivated tomato and its wild relatives was constructed from 725 tomato data, containing important genes not found in the reference, such as *Hcr9-OR2A*, *I2C-1* and *Pto*. An analysis of PAVs showed that gene loss from the domestication process was lower than that from the improvement process [47]. In cultivated soybean, a total of 723,862 PAVs were identified from the graph pangenome, representing approximately 16% of a single genome. These variants are related to soybean grain gloss variation and to the differentiation of wild species and cultivated species [57]. In another case, by mapping 354 sorghum variety sequences from different genetic backgrounds to the sorghum pangenome, approximately 2 million SNPs were identified, of which 398 were associated with agronomic traits and 1,788 were involved in the drought stress response [59]. Multi-population pangenomics uses the high-quality TGS-genomes of multiple representative varieties to further help people decode the genetic mechanism of the differentiated phenotypes of polyploid plants and accelerate the breeding process of plants [57, 75, 76]. Another important improvement is the creation of a “super pan-genome” for plants, which extends the concept of the pan-genome to the genus level, promotes the analysis of scientific issues such as inter-individual gene exchange and genome evolution after polyploidy, and provides a theoretical basis for the rational utilization and preservation of wild germplasm resources [76, 82–84]. A recently published article on the pangenome of tomato extends the concept of pangenome to a new field [85]. This article demonstrates the significant benefit of pangenomic genetic diversity in retrieving the “missing heritability” from three aspects. A large number of SVs, their nearby SNPs, and indels were found to exhibit strong incomplete linkage disequilibrium. It also showed that employing pan-variations increased the estimated heritability of tomatoes by 24% and found two possible SVs that are substantially linked with soluble solid content and may be employed in future marker-assisted selection. This study shows how pangenomic variations can enhance GWAS’s capacity for detection and lays the groundwork for the use of SV in the development of molecular markers in the future.

### The pangenomic model of animals differs from that of plants due to their unique genetic characters

The number of published articles focusing on the animal pangenome is much lower than that for plants, and are mainly related to the generation of mutations and population genetic processes [53]. In general, the main focus in pangenome research is the variation in the genome, which usually appears in the form of mutations. With the exception of neutral mutations (affected by genetic ticket changes), other types of mutations can usually only be fixed or eliminated under selection pressures. Thus, when performing pangenomic analysis, attention should be given to the rate of variation, the effective population size and the proportion of neutral SVs in different mutants. Second, abundant transposable elements in plants have been proven to produce rich species diversity by mediating sequence rearrangement and regulating the expression of nearby genes [86, 87]. Compared with animals, plants have generated numerous variations after multiple polyploidization events during their evolution [88, 89], and have also generated more strains, more complex agronomic traits and larger effective population sizes [53, 90]. For the above reasons, the study model of animal pangenomes is different from that of plants.

To date, the pangenome of animals mainly uses large-scale comparative genome to reveal variants in animal genomes or to search for specifically expressed genes related to animal origins, evolution and phenotypes. For instance, genome comparisons of 44 ruminants [30], 6 ticks [91], 16 *Heliconius* species [92] and 11 flatfish species [93]. Only a relatively small quantity of studies have linked these variants to biological resistance and adaptability [94, 95]. The more typical case is the construction of the pangenome of Mediterranean mussels [62]. This study showed that an average of 4,829 (8.01%) protein-coding genes and 3,744 (5.12%) noncoding genes were missing per re-sequenced individual. Further analysis confirmed the complex pangenome structure of purple mussels, among which nonessential genes were mainly related to hemizygous regions of the genome (~ 580 Mb sequence missing in the reference genome). These genes are highly expressed in the pathways of apoptosis, stress resistance and immune response, suggesting a relationship between mussel pan-sequences and biological adaptation, but this relationship remains to be tested due to the lack of variation and adaptive information on phenotypes across geographical regions.

## Domestic animal pangenome studies reveal the widespread hidden genetic variations in different populations

**Fig. 7:**
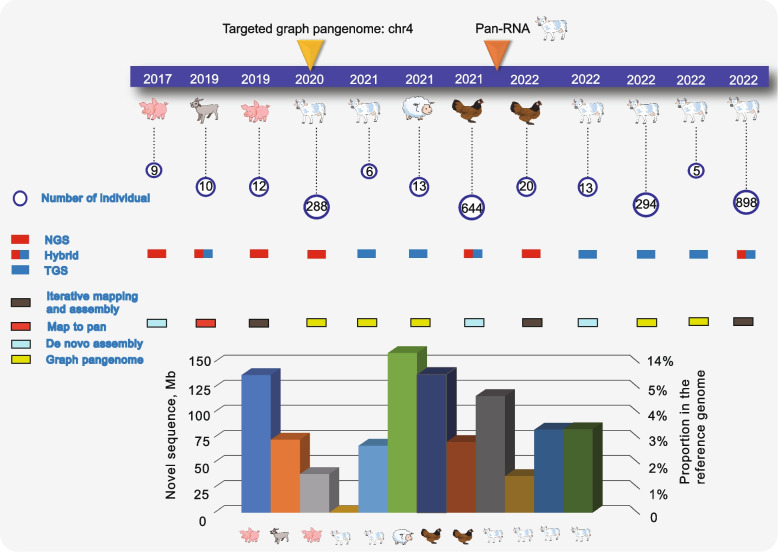
Overview of the pangenome in livestock and poultry. The numbers in the circles are the individuals involved in building the pangenome. The sequencing technology and the method of constructing a pangenome are indicated in different colors. The bottom bars show the number and proportion of novel (non-reference) sequences in each species

Due to the particularity of the geographical location and the domestication mode of livestock and poultry, the difficulty of ideal sample collection has increased. Domestic animal pangenome research has slowed down as a result. Among them, pigs were the first to be the subject of pangenomics. In the existing cases, the proportion of new sequences found was 1.3%–14.9% (Fig. 7), with a large number of biologically significant functional genes. These genes are mainly enriched in relation to the immune responses of various species, indicating that domestic animals can improve their resistance through these genes to better adapt to extreme environments such as cold and high temperatures [91, 96]. Additionally, the pangenome reference model has a better ability to discriminate SVs by validation of different WGS data [14]. Many of the SVs identified from this reference model were associated with important biological phenotypes of livestock or poultry as well as with domestication improvements [45, 97]. The biological variants to be revealed can help us to deeply understand the hidden mechanisms underlying different phenotypes, and can facilitate the full use of these genetic resources. The SV sets and novel sequence variations built in light of the pangenome break the long-held restriction of using SNPs and indels for hereditary examination, and will give another strategy to dissecting the hereditary structure of the livestock and poultry breeds in the world.

### The pangenome of pigs

The first related article was published in 2017 [98], through compared the genomes of nine pig breeds from different ecological regions in Asia and Europe and found a large number of new genomic variations and missing sequences of 137.02 Mb. The total variation of these sequences in Chinese pigs was significantly greater than that in European pigs. In 2019, Tian et al. [50] constructed the first pig pangenome (containing nonreference sequences of 72.5 Mb, or 3%) using 12 de novo pig genomes. Furthermore, 87 resequencing data were compared with the pangenome, which revealed a high frequency of approximately 9 Mb of pan-sequences in Chinese pigs, covering a regulator of adipose lipolase, tazarotene-induced gene 3 (TIG3), which is specifically expressed in Chinese pig breeds and leads to fat deposition. At the same time, the content of these generic sequences varied greatly in different sexes and contained a large number of SNPs. The construction of the pig pangenome highlights the genomic variation that is not fully displayed by the reference genome. Studying genetic variation at the pangenome level can help identify those mutations neglected in the past and can facilitate genome downstream analysis.

### The pangenome of goats

For goats, only one cross-species pangenome consisting of goats and their sibling species was reported in 2019 [52]. This pangenome contains the 38.3 Mb sequence missing from the reference ARS1, and only 1% of the sequences are widely present in individuals. Most pan-sequences were identified as PAVs, for example, as an insertion of 18.8 kb was found on chr1, which partially covers the gene region of the pelanin-like gene. Validation with the transcriptome and resequencing data showed that both SNP calling and transcriptome mapping rates were significantly higher than when using ARS1 as the reference, confirming the reliability of the pangenome. However, since this pangenome was constructed on the basis of the genomes of eight related goat species, it would mask some true pan-sequences from the goat population. Second, although this method can identify more generic sequences, the structural composition analysis of generic sequences is not ideal (e.g., the identification of TEs and segmental duplications).

### The pangenome of sheep

To further characterize the SVs in domestic sheep, Li et al. [99] constructed the first graph pangenome of sheep from 13 representative sheep breeds with 26 haplotype-resolved assemblies. This pangenome size is 2.75 Gb and contains 137.7 Mb (5.6%) nonreference sequences. Based on this pangenome for SV typing of 687 resequenced samples, 115,089 SVs were identified, and 5.3% of the high-frequency de novo SVs may be related to the domestication of sheep. In addition, genome-wide selection signal methods were used to prove that 865 population-stratified SVs can affect the expression of 304 genes related to different phenotypes and production traits in sheep, including wool type (IRF2BP2, FGF7) and tail morphology (HOXB13). Further validation in populations of sheep with different tail types revealed that the selected de novo SVs around the SNPs are involved in the formation of the fat tail phenotype and identified putative pathogenic mutations in the *HOXB13* gene that cause the long tail. These results prove that graph pangenome reference patterns are more conducive for mining the hidden structural variants and for identifying causal mutations and the potential effects of these SVs on phenotypic changes.

### The pangenome of cattle

Many studies have made efforts to improve the pangenome of domestic cattle [100–102]. The first pangenome of cattle was released in 2020. It is a graphic genome built on the frequency of 288 alleles among four cattle breeds, which includes 243,145 variants. Simulation analysis of the haplotype mapping ratio of these individuals showed that the reading error rate of the mapping based on the graph pangenome was 30% lower than that of linear reference [103]. Because the cattle of these four breeds belong to different groups genetically, this result also illustrates the possibility of establishing a universal bovine pan-gene map. On the other hand, owing to the lack of both long-read and large-fragment SVs data, pangenome graphs based on SNPs and indels obtained from short reads and single references may ignore many new and large variations.

In another case, a total of 70.3 Mb of nonreference sequences (containing 76% repeating elements) were detected from 5 bovine de novo genomes, most of which were from the yak genome [101]. Transcriptome and gene predictions showed that these sequences contain numerous functional sites involved in important pathways, such as the immune response and lipid metabolism. Otherwise, there were differences in the number of transcripts found and in the differentially expressed genes across the breeds, indicating that the reference genome contributes rather differently to individuals at different genetic distances during genetic analysis.

A recent study integrated five genomes and NGS data from 294 cattle into a graphical genome containing global cattle diversity [51]. A sequence of 116.1 Mb (4.2%) was revealed that is currently lacking in the bovine reference genome. The newly assembled genomes of two African breeds fill the current blank in the lack of available high quality reference genomes for African cattle. By comparing different datasets, it was confirmed that the graph genome, which could represent the genetic diversity of cattle breeds throughout the world, has more power for SV calling and prediction of novel functional regions than linear references. Importantly, it was the first to provide direct evidence that pangenome can facilitate the downstream analysis of genomics. It additionally affirmed that the development of ideal pangenomes ought to involve excellent and complete genomes as skeletons, like the utilization of TGS and telomere-to-telomere (T2T) assemblies. Furthermore, a study using 898 cattle generated the biggest cattle pangenome (57 breeds) and found 83 Mb of non-reference sequences. It provides a novel approach for studying the pedigree composition and accurate identification of cattle breeds around the world, which overcomes the previous limitations of SNP-based genetic analysis [102].

Currently, published bovine pangenome studies have mined and verified the possibility of the pangenome from multiple perspectives, such as SV calling and assembly quality. It also showed that the pangenome was a valuable resource for studying species diversity, domestication, and evolutionary history. High-accuracy genomes and comprehensive variant sets obtained from the construction of the pangenome can offer available reference genome resources for some excellent cattle varieties and enhance the omics research of this breed worldwide, contributing to an expanded understanding of how trait breeding and introgression shape the bovine genome and their native fitness.

### The pangenome of chickens

The first pangenome of domestic chickens was published in 2020. It was constructed using an iterative mapping and assembly approach, with WGS data from 664 individuals and the reference genome GRCg6a [45]. To better explore PAV, the authors compared 268 WGS data from individuals to the pangenome and identified 15,205 (76.32%) core genes and 4,738 variable genes. Further analysis revealed that hybridization affected PAV gene content more than genetic drift did during chicken domestication and improvement. In addition, the PAV frequency of promoter regions changed significantly during breeding, and 81 traits, including chicken carcass composition and meat quality, were associated based on the results of the PAV-GWAS analysis. The growth traits of chickens were mainly related to the deletion of the IGF2BP1 promoter region on chromosome 27.

A later study identified 159 Mb of new sequences by comparing 20 de novo assemblies containing 1335 protein-coding genes and 3011 long noncoding RNAs [97]. New sequences and genes are mainly distributed in regions with high recombination rates, and the elevated substitution of most new genes is threefold greater than that of known genes, which greatly improves the average substitution rate of the chicken genome. Consistent with other species, approximately 13.1% of the new genes act as housekeeping genes, while the vast majority of the new genes are concentrated in basic biological pathways such as immune response, metabolism and disease. Advances in the chicken pangenome have provided new insights into the genetic structure of different breeds and the relationship between phenotypes and genes. Moreover, these advances will further promote avian evolution research, functional genomics and the targeted breeding of specific traits in chickens.

## Challenges in livestock and poultry pangenome

With the innovation of TGS, assembly algorithms and software, it is possible to explore large fragments of genomic variation and repetitive regional structural features while overcoming the high error-tolerance rate [23, 104–107]. The genomes of domestic animals are also constantly being improved and updated, which is of great help to the accurate analysis of the pangenome. For example, the newly released version of the goat genome, Saanen_v1, reported and corrected for assembly errors in ARS1, improved the assembly of the X chromosome, and generated the first goat Y chromosome assembly [25]. In the porcine reference genome (ssrofa11.1) published in 2017, a total of 15,544 human homologous genes and 15,958 highly linear conserved genes were annotated, which is 2,625 and 4,297 more, respectively, than those annotated by Sscrofa10.2 [26]. Approximately 19% of new genes were found in the cattle reference genome ARS-UCD1.2, and the missing centromere and telomeric repeats in UMD_3.1.1 were also observed on nine chromosomes (5, 6, 8, 10, 13, 14, 16–18) [27].

Perhaps the foremost challenge presented by the advent of this new idea is determining how best to create a complete pangenome under the current conditions. Although pangenomics, as an emerging research field, has the advantage of making up for past linear references and detecting genetic variation from a larger genome range, there are still the problems of high visualization difficulty and heavy requirements of algorithms and analytical methods [108]. Fundamentally, the ideal state of pangenome analysis is to reach a “complete” level, that is, to compile all functional originals and sequences. When used as reference coordinates, were able to represent different species, niches, organizations or the same species under different taxa of genome information. Such pangenome data has the characteristics of “big data” such as large volume, complexity and rapid output, which requires powerful computing and storage capabilities [109]. These problems impose relative limitations on the development of the species pangenome.

In addition to the hardware requirements, most studies do not adopt uniform standards for defining sequence similarity, and there is a lack of normative and formal construction procedures. In pangenome studies, the selection of alignment algorithms and how to define or distinguish between orthologous and paralogous genes has a large impact on the core and variable genome content. For instance, Li et al. [69] defined sequences with length > 100 bp and < 90% identity as missing, while Sherman et al. [75] used ≥ 50% of the contig length and ≥ 80% identity to screen novel sequences distinct from the human reference genome. This led to differences in the new sequence content identified by the two. Ruperao et al. [46] used more stringent screening criteria (> 90% coverage and greater than 90% identity) to ensure the non-redundancy of the new sorghum sequences. In the pig pangenome, sequences with < 90% identity and a size of ≥ 300 bp were identified as differential sequences [50]. Beyond that, pangenome construction involves complex processes such as assembly, annotation and alignment, which are matched by numerous software or algorithms, and different combination strategies have different impacts. Therefore, how to formulate the optimal strategy to ensure the best results is a priority to be considered before analysis.

Meanwhile, it was found that the proportion of variable parts of the livestock and poultry pangenome (1.3% [52]–14.9% [97]) was lower than that of plants (8.1% [110]–69.4% [58]), and most of the sequences contained highly repetitive sequences. Nevertheless, NGS technology can only read 100–400 bp reads, which is unfavourable for the identification of repeat regions. This requires the use of some high-quality assemblies to find more hidden genomic variations. Although many high-quality reference genomes have been produced, it is notable that population-level long-read genomic data from certain good local breeds are still lacking, particularly for breeds with distinguishing traits in domestic animals like goats, cattle, and pigs.

Some studies shown that the selection of different references results in inconsistent fragment difference information [30]. Combined with plant research, these results suggest that the ploidy levels, kinship distance, wild or breeding species, and domestication history will directly affect the nonessential gene content. This affects the resolution and integrity of pangenomic assays. Consequently, how to select materials to obtain a pangenome at higher resolution and with greater completeness is also a problem. For domestic animals, the selection of populations should also avoid some high inbreeding or highly homogeneous individuals.

Another important issue is that the numerous data generated in current pan-genome studies are not effectively utilized. The current pangenome of most species are constructed based on high-quality de novo genomes obtained using PacBio or Nanopore sequencing technology. However, the majority of these genomic data are limited to exploring the SV landscape of multi-populations and the impacts of this extensive variation on gene expression [58], phenotypic changes [99], and adaptability in species [95]. Only a few studies have expanded on the subject to investigate the role of SV in gene regulation [49], heritability recovery [85], and chromatin rearrangement [61].

In fact, these high-quality assemblies, well-established annotations and SVs are excellent models for studying chromosome evolution, ncRNA biological function, and epigenomics. In particular, large inversions in SV have been shown to have a very important evolutionary role in that they can inhibit recombination, giving them a significant role in the evolution of sex-chromosomes, speciation, and local adaptation [111]. In the case of the Y chromosome, it evolves as an asexual genetic unit after being genetically separated from the X by inversions. The Y then degenerates as a result of a number of evolutionary mechanisms, including Muller’s ratchet and harmful mutations that link to advantageous mutations to arrive at fixation [112]. But pangenomics investigations of chromosomal sequence composition, structural evolution, and non-coding RNA function have not yet been reported. Research in this area is mainly through the acquisition of a single representative animal’s de novo genome, such as the duck [113], horse [114], goat [115] and sheep [116]. The limitations of this application may be related to the following aspects: the great difficulty of obtaining a perfect genome mentioned above; the large and numerous repetitive sequences that make it challenging to obtain a complete annotation; and the lack of ideal software for pangenomic analysis, especially for repetitive changes in large structural variants [117]. While the pangenome integrates the genetic information of multiple varieties, analyzing the structure and composition of chromosomes at this level can provide a more comprehensive understanding of their evolutionary characteristics and mechanisms. Therefore, it would be wise to discuss new genetic information recording methods and analytical technique to consider how to effectively use this high-quality genomic data in the future to investigate new areas like sex-chromosome evolution and others not involved in current pangenomics research.

## Conclusions and future perspectives

In conclusion, under different selection pressures, such as natural selection, artificial selection and balanced selection, domestic animals have developed personalized phenotypic changes while adapting to various environments. These resources are excellent materials for pangenome studies. Moreover, recent advances in human and crop pangenomes also suggest that SVs in the pangenome are important genetic resources to explore the processes of species domestication, migration, and biological adaptation. Improvements in NGS, TGS, genomic analysis pipelines and algorithms also pave the way for the implementation of pangenome projects in domestic animals.

**Fig. 8:**
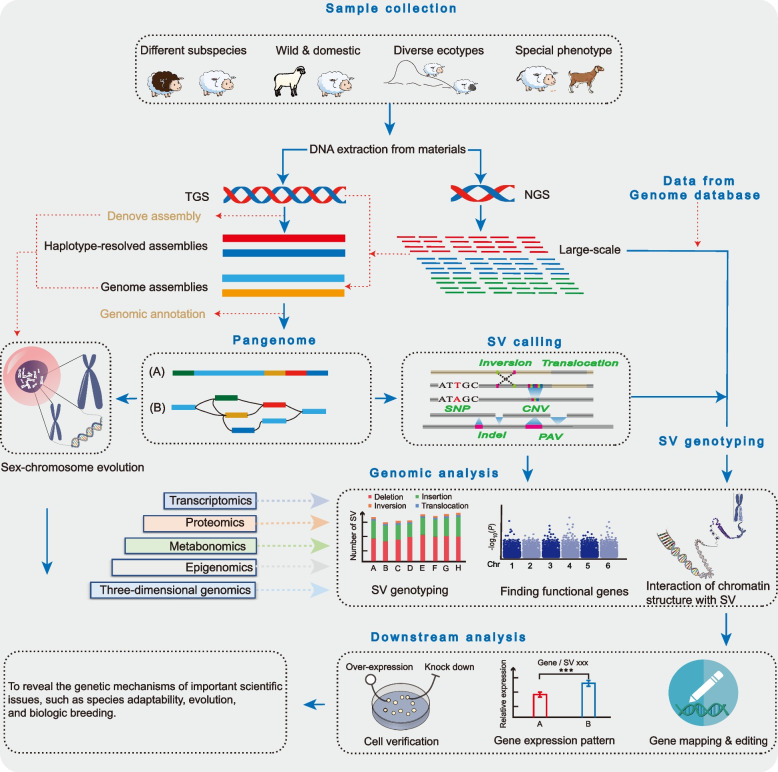
The synopsis of the model for future research on the pangenomes of livestock and poultry. In the future, livestock pangenome research should effectively combine TGS and NGS data. First, representative samples should be selected to extract high-quality DNA for TGS or NGS sequencing. Second, the high-quality genomes or haplotype-resolved assemblies are generated to construct the graph pangenome, then performs SV detection and identification. Thirdly, newly generated or existing NGS data in the database are compared with the pangenome for genotyping. Combining with multi-omics data, the SV affecting the important economic traits of livestock and poultry will be mined and verified from multiple angles and levels. Finally, the molecular markers found should first be validated at the cellular level. If possible, gene editing technology can be used to verify the effect of the functional gene on phenotype at the individual level

In future studies, it may be possible to consider using hybrid assembly to build the pangenomes of livestock and poultry (Fig. 8). This approach can economically and efficiently analyse genomic variation and make it possible to mine rare genes by integrating existing WGS data and TGS de novo components of some individuals. In terms of this approach, the high-quality genome obtained by TGS can capture the signals of telomeres, centromeres and complex regions with high repetition. More complete chromosomes can ensure the accuracy of genome mutation regulation and can eliminate most of the false-positives. Moreover, pangenome integration de novo assembly and/or haplotype resolved assembly can more likely distinguish variants. On this premise, the investigation of variation at the single nucleotide level utilizing reference genome-free strategy is key to determine breakpoints and correlation analyses. Many studies have confirmed the accuracy and sensitivity of pangenome calling SVs, and when using these high-quality genomes to generate SV sets, these SVs can be genotyped using NGS data. Further characterising population structure, phylogeny, selection signals and GWAS analysis of the typing results to identify the SVs related to important economic traits of livestock and poultry and using as those traits as molecular markers to assist in biological breeding will further promote the healthy development of the seed industry.

Another important concern is that current pangenome research on livestock and poultry is mainly focused on the coding region of the genome, and there is also a lack of transcriptome pangenome studies, which have only been implemented for a few species [118]. Moreover, ncRNA and mitochondrial DNA are also important resource for studying the historical evolution, selection and genetic differentiation of populations. And the question of how the sex-chromosomes evolve is also one worth exploring. These aspects have not yet been reported in current research. Finally, the vast majority of eukaryotic pangenomes, including those of plants, are restricted to the “species” level, and only in prokaryotes have pangenome studies been extended to higher taxa such as “genera”. Therefore, future pangenome research on livestock and poultry can include studies on noncoding region DNA, RNA and mitochondrial DNA. New genomic technologies such as T2T will make it more possible to explore the complex structure of the sex-chromosome in livestock and poultry, which will bring new understanding to the theoretical paradigm of their evolution. Therefore, when conducting pangenome analysis, we should make full use of the resulting high-quality assemblies, combine them with other omics, and break through the existing application limitations to expand the analysis of hot scientific issues in multiple fields. Additionally, it is necessary to break the current pangenomic model of intraspecies sequences or gene sets and create a pangenome at the genus level that includes intraspecies genomes or genomic variants. These findings will further expand the depth of the domestic animal pangenome and help us deeply analyse the origin, domestication and adaptive mechanisms of livestock and poultry. In the future, sequencing costs will gradually decrease, and computing resources will gradually expand, which will promote trans-genus pangenomes and help us understand the fundamental problem of the relationship between genes and species origin.

## Data Availability

Not applicable.

## Abbreviations

CNVs: Copy number variations
gCNVs: Gene copy number variations
indels: Small insertions and deletions
NGS: Next-generation sequencing
PAVs: Presence/absence variants
SNPs: Single nucleotide polymorphisms
SVs: Structural variants
TGS: Third-generation sequencing
TEs: Transposable element
T2T genome: Telomere-to-telomere genome
